# Is preventable sight loss truly preventable? An exploration of a public health indicator for sight loss due to age-related macular degeneration in England

**DOI:** 10.1038/s41433-022-01933-7

**Published:** 2022-02-23

**Authors:** Kelsey Brown, Catey Bunce, Oluwaseun Onabanjo, Stacey A. Strong, Praveen J. Patel

**Affiliations:** 1 Kings College London School of Population Health and Environmental sciences, Faculty of Life Science and Medicine, London, SW10 0LB UK; 2 Research Data & Statistics Unit, Royal Marsden Clinical Trials Unit, Royal Marsden NHS Foundation Trust, London, UK; 3 Kings College London School of Population Health and Environmental sciences, Faculty of Life Science and Medicine, London, UK; 4 NIHR Biomedical Research Centre at Moorfields Eye Hospital NHS Foundation Trust and UCL Institute of Ophthalmology, London, UK

**Keywords:** Macular degeneration, Risk factors

## Abstract

**Background:**

Age-related macular degeneration accounts for the majority of severe sight impairment and sight impairment registration and certifications in adults in the UK [1, 2]. Whilst these treatments are effective in arresting nAMD progression, there is currently no treatment for GA [1, 3, 4].

**Methods:**

This paper provides an update to the data collected by Bunce et al. [3] and details the number of people certified together with incidence rates for the various types of AMD by: sex, sight impairment status, and for all ages using the 2016/2017 and 2017/2018 CVI due to AMD data for England from the Moorfields Eye Hospital, supplemented with 2017–2018 PHOF indicator 4.12i/E12a data. The study population includes individuals of all ages in England who were newly certified with visual impairment due to AMD.

**Results:**

Between 2016 and 2017, CVIs due to AMD totalled to 11,215; between 2017 and 2018, CVIs due to AMD totalled to 10,914. The PHOF indicator 4.12i/E12a assessed showed that overall rates of AMD certifications have steadily declined in England from 131.5 per 100,000 in 2010/2011 to 106.7 per 100,000 in 2017/2018.

**Conclusion:**

As treatment is available for nAMD, a reduction in nAMD certifications could be expected; however, growth of the elderly population in England combined with there currently being no treatment available for GA means AMD certification rates should be increasing. Therefore, it is postulated that not all cases of AMD are being certified and registered with some likely going undiagnosed.

## Introduction

Age-related macular degeneration (AMD) is the primary cause of ocular morbidity, vision impairment, and blindness in elderly populations of high-income countries [1, 2, 5–7]. AMD accounts for the majority of severe sight impairment (blind) and sight impairment (partially sighted) registration and certifications in adults in the UK [1, 2].

Internationally recognised definitions of late or advanced AMD consist of two main forms with differing pathophysiology and prognosis [6]. Geographic atrophy (GA) causes sight loss over a period of months to years and is characterised by atrophy of photoreceptors, retinal pigment epithelium, and choriocapillaris; wet AMD, also referred to as neovascular AMD (nAMD) or exudative AMD, is characterised by blood vessel leak leading to rapid sight loss often over days or weeks [3, 6–8].

Thus far, only nAMD is treatable. The National Institute for Health and Care Excellence (NICE) recommends pharmacological treatments with agents which block the action of vascular endothelial growth factors given by intravitreal injection. Ranibizumab (Lucentis^®^; Genentech-Roche/Novartis) was approved in August 2008 [4, 9, 10] and aflibercept (Eylea^®^, Regeneron Pharmaceuticals/Bayer) was approved in July 2013 [4, 11]. Bevacizumab (Avastin^®^, Genentech-Roche/Novartis) has been used successfully to treat nAMD in specific cases. It is chemically related to ranibizumab and significantly cheaper, however, it is not currently approved by NICE and therefore only used ‘off-label’ [10].

Whilst these treatments are effective in arresting nAMD progression, there is currently no treatment for GA [1, 3, 4]. Supportive low vision services provide visual aids and advice regarding smoking cessation and increased dietary intake of vitamins and antioxidants [3, 4].

In England, registration for sight loss is achieved following diagnosis and completion of a Certificate of Vision Impairment (CVI) form by a consultant ophthalmologist. Patients are certified by sight impairment status as either sight impaired (SI) or severely sight impaired (SSI) [3]. The main cause of vision loss is selected from a list of diagnoses. The various options for AMD include: nAMD, GA, mixed AMD, and Multiple pathology including AMD (whereby an ophthalmologist is unable to define a single cause for sight loss in one or both eyes) [3].

After certification, a copy of the CVI is sent to the Royal College of Ophthalmologists c/o Certifications Office based at Moorfields Eye Hospital, London (“Moorfields Eye Hospital”).

This is used for anonymised data analyses, which are incorporated into the Public Health Outcomes Framework (PHOF) by Public Heath England (PHE) under the indicator for preventable sight loss (4.12/E12) on the PHOF website: indicator group E. Healthcare and premature mortality, number E12 and ID 41201–41204 with four sub-categories:

- 4.12i/E12a New CVIs due to AMD aged 65+, per 100,000 population
- 4.12ii/E12b New CVIs due to glaucoma aged 40+, per 100,000 population
- 4.12ii/E12c New CVIs due to diabetic eye disease aged 12+, per 100,000 population
- 4.12iv/E12d New CVIs, per 100,000 population

Another copy of the CVI is sent to the patient’s local authority that offers a needs assessment and adds the patient to the local registry of persons with sight impairment. Certification and registration forms are only completed by patient choice and consent (not statutory), but the forms are required to access social service benefits [3].

The introduction of effective treatments for nAMD, together with certification and registration of sight loss, has allowed for more efficient planning of healthcare provision for AMD patients, with effective medical and social support to hopefully slow disease progression and ease disease burden [4, 5, 8].

However, AMD impacts quality of life significantly with increased risk of disability and morbidity, for example, mental health issues. It also places a huge burden on healthcare and the economy [12, 13]. The contributing risk factors include: gender, health inequalities, deprivation, smoking habits, diet, obesity, and most importantly age [14]. A statistically significant correlation was identified between visual acuity and the Index of Affective Suffering score [15]. This, coupled together with the growing and ageing population in England, will likely increase the burden of disease of AMD [1, 2]. When viewed within the context of the World Health Organization’s initiative for the elimination of avoidable blindness by 2020, [16] it is now more important than ever to assess incidence rate (IR) trends and public health impact of AMD with a view to progressively tackle the problem.

Furthermore, there are suggestions that CVI and registration data for SI and SSI in England may be incomplete. With regards to AMD, CVI data only provides incidence of new certifications, not prevalence or incidence of the disease. This is because it excludes those patients who remain undiagnosed or refuse certification. In addition, Local Authority register data for blind or partially sighted people does not include cause of sight loss and excludes those who choose not to be registered or those with AMD that do not qualify for registration [1, 5]. The actual burden of disease may therefore be underestimated when using CVI data alone, or when supplemented with blind or partially sighted registration data.

This is supported by Bunce et al., which describes patients who receive or anticipate treatment for nAMD being three times less likely to be certified as treatment is available; whereas those with GA and no treatment available are more likely to certify in order to facilitate access to long-term health and social care [3]. Bunce et al. [3] and a review by Owen et al. [1] suggested that a trend of higher certification rates for all types of AMD would continue from 2007 to 2012 and beyond.

PHOF grouped all cases of AMD (whether that be nAMD, GA, or mixed) under the indicator of preventable sight loss due to AMD. As discussed, however, only nAMD is treatable at this present time and hence preventable, therefore the labelling of AMD by PHOF can be argued a misnomer. In addition, the PHOF indicator 4.12i/E12a only provides IRs for those over 65 years old.

This paper aims to update the data collected by Bunce et al. [3] and detail the number of people certified together with IRs for the various types of AMD by: sex, sight impairment status, and for all ages using the 2016/2017 and 2017/2018 CVI due to AMD data for England from the Moorfields Eye Hospital, supplemented with 2017–2018 PHOF indicator 4.12i/E12a data. The actual IR of preventable sight loss due to AMD will be subsequently postulated by calculating the IR of nAMD for 2017/2018. The PHOF data will be used to examine trends over the years and regional variability across England in certification rates for AMD.

## Methods

This research was a secondary data analysis of the 2016/2017 and 2017/2018 CVI due to AMD data for England provided by the Moorfields Eye Hospital and the 2017–2018 PHOF indicator 4.12i/E12a, preventable sight loss due to AMD.

The study population includes individuals of all ages in England who were newly certified with visual impairment due to AMD.

### Data collection and statistical analysis

The 2017–2018 PHOF indicator 4.12i/E12a measures count and crude rate of preventable sight loss due to AMD; defined as new certifications of visual impairment due to AMD in those aged 65 years and over in England, between 01 April 2017 and 31 March 2018 [17]. It does not differentiate the various types of AMD. The numerator count includes sight loss due to AMD as the main or contributory cause as documented on the CVI. The denominator is from 2017 to 2018 mid-year population estimates, aggregated for persons 65 years and over [18].

The crude rates are reported in rate per 100,000 population [17, 18]. The crude rate 95% confidence interval (95% CI) is calculated using Byar’s method, described elsewhere [19, 20]. These data were analysed by the PHE intelligence division from anonymised data provided by the Moorfields Eye Hospital and the Office of National Statistics (ONS). These data are regularly reported and readily available through the PHOF website [17]. The PHOF data will be used to examine trends between 2010 and 2018 and identify regional variability across England in certification rates for AMD.

The 2016/2017 and 2017/2018 CVI due to AMD data was provided by the Moorfields Eye Hospital. As previously discussed, a copy of every CVI is sent to the certifications office, which is anonymised and converted into an electronic version to store on a large database. The CVIs are divided into nAMD, GA, Mixed AMD, or Multiple causes including AMD, or unknown. The time span of data was between 01 April 2016 to 31 March 2017 and 01 April 2017 to 31 March 2018. This dataset was anonymised and contained all ages. The certifications office functions under the auspices of the Royal College of Ophthalmologists (RCOphth). The RCOphth and Department of Health and Social Care have named the certifications office at Moorfields as the supplier of data for PHOF preventable sight loss indicator 4.12/E12 [17].

This dataset included sex, age, sight impairment status, type of AMD, and date of certification.

The patients were divided into four types of AMD:

1. Dry AMD or GA
2. Wet AMD (nAMD)
3. Mixed AMD
4. Multiples causes including AMD

The types of AMD were then cross tabulated against:

- Sight impairment status: Sight Impaired (SI), Severely Sight Impaired (SSI)
- Age groups: Under 50 and quinary age groups between 50 and 90 years of age
- Sex: Male, Female.

This dataset allowed for the calculation of age and sex specific IRs of AMD and age specific IR estimates for the different types of AMD; using the ONS mid-year population estimates for the corresponding year as the population at risk, aggregated by sex and age groups [18]. IRs and their 95% CI were calculated using Byar’s method and expressed in rate per 100,000 population, described elsewhere, [20] using the Open Source Statistics for Public Health tool [21]. This allowed for a wider range of ages to be represented compared to the single grouping in PHOF i.e. 65+ years. Robust estimates of IRs in older ages are particularly important as that is where AMD is more prevalent [5].

## Results

Between 01 April 2016 and 31 March 2017, 23,453 CVIs were received. Table 1a shows that the CVIs due to AMD amounted to 11,215 of which 6343 (56.55%) were classified as SI and 4728 (42.15%) as SSI. The remaining 144 CVI forms had no sight impairment status recorded.

**Table 1:** (a) Sight impairment status categorised by type of AMD from 2016/2017 CVI due to AMD data in England. (b) Sight impairment status categorised by type of AMD from 2017/2018 CVI due to AMD data in England.

Sight impairment status	Dry (or GA)		Wet (nAMD)		Mixed AMD		Multiple cause incl. AMD		Total
	*n*	% of T	*n*	% of T	*n*	% of T	*n*	% of T	T2
(a)									
SSI/Blind	1893	40.87	1409	44.99	639	36.06	786	47.21	4728
SI/Partial	2679	57.84	1686	53.83	1110	62.64	856	51.41	6343
Total T	4632		3132		1772		1665		11,215
(b)									
SSI/Blind	1913	42.59	1370	45.18	729	39.9	783	50.55	4800
SI/Partial	2536	56.46	1636	53.96	1082	59.22	755	48.74	6017
Total T	4492		3032		1827		1549		10,914

(a) T includes unknown sight impairment status.

(b) T2 includes unknown type of AMD.

*AMD* age-related macular degeneration, *CVI* certificate of vision impairment, *GA* geographic atrophy, *nAMD* neurovascular age-related macular degeneration, *SI* sight impaired, *SSI* severly sight impaired.

Between 01 April 2017 and 21 March 2018, 22,844 CVIs were received. Table 1b shows of these 10,914 were CVIs due to AMD with 6017 SI cases (55.13%) and 4800 SSI cases (43.98%). Ninety-seven CVI forms did not have the sight impairment status recorded.

In both datasets (Table 1a, b) GA made up the majority of certifications in 2016/2017 and 2017/2018, 4632 (41.30%) and 4492 (41.16%) respectively. Between 2016 and 2018 there was a reduction in the number of certifications overall and in most types of AMD except CVIs due to Mixed AMD which increased.

The total amount of certifications for sight loss due to AMD in England increased as age increased, with the highest IR in those aged 90 years and over in 2016/2017 and 2017/2018, at 691.88 per 100,000 (95% CI 668.7, 715.6) and 672.80 per 100,000 (95% CI 650.1, 696.0) respectively (Table 2a, b). More women (around 66%) were certified with vision impairment than men in both 2016/2017 and 2017/2018. The incidence of certification due to AMD positively correlated with increasing age in both sexes, with the highest IR seen in females aged 90 years and over in 2016/2017 and 2017/2018, 694.34 per 100,000 (95% CI 666.6, 722.9) and 677.96 per 100,000 (95% CI 650.6, 706.1) respectively. Over 80% of CVI due to AMD certifications in 2016/2017 and 2017/2018 were accounted for by those aged 80 years and over, compared to less than 4% in those under 70 years old.

**Table 2:** (a) Age- and sex-specific incidence rates for CVIs due to AMD, all ages, in England 2016/2017, per 100,000 population. (b) Age- and sex-specific incidence rates for CVIs due to AMD, all ages, in England 2017/2018, per 100,000 population.

Age category	Female				Male				Sex N/A	Total (male and female) T2			
	*n*	IR	95% CI lower limit	95% CI upper limit	*n*	IR	95% CI lower limit	95% CI upper limit		*n* (%T)	IR	95% CI lower limit	95% CI upper limit
(a)													
0–49	22	0.13	0.1	0.2	*?	*	*	*		32 (0.29%)	0.09	0.1	0.1
50–54	12	0.61	0.3	1.1	*?	*	*	*		21 (0.19%)	0.54	0.3	0.8
55–59	19	1.11	0.7	1.7	13	0.79	0.4	1.3		32 (0.29%)	0.95	0.6	1.3
60–64	60	4.02	3.1	5.2	41	2.85	2	3.9		101 (0.90%)	3.46	2.8	4.2
65–69	125	8.01	6.7	9.5	79	5.37	4.3	6.7		204 (1.82%)	6.73	5.8	7.7
70–74	320	25.73	23	28.7	218	19.17	16.7	21.9		538 (4.80%)	22.59	20.7	24.6
75–79	780	80.56	75	86.4	411	49.65	45	54.7		1192 (10.63%)	66.37	62.7	70.2
80–84	1519	199.57	189.7	209.9	834	142.75	133.2	152.8		2355 (21.00%)	175.04	168	182.3
85–89	2169	420.16	402.7	438.2	1166	359.86	339.5	381.1		3337 (29.75%)	397.17	383.8	410.9
90+	2364	694.34	666.6	722.9	1009	684.62	643	728.2		3375 (30.09%)	691.88	668.7	715.6
Age not stated	21				*/n/a					28 (0.25%)			
Total T	7411				3793				11	11,215			
% of T	66.08%				33.82%								
(b)													
0–49	10	0.06	0	0.1	11	0.06	0	0.1		21 (0.19%)	0.06	0	0.1
50–54	12	0.61	0.3	1.1	6	0.31	0.1	0.7		18 (0.16%)	0.46	0.3	0.7
55–59	18	1.02	0.6	1.6	23	1.34	0.8	2		41 (0.38%)	1.18	0.8	1.6
60–64	39	2.57	1.8	3.5	30	2.05	1.4	2.9		69 (0.63%)	2.31	1.8	2.9
65–69	121	8.11	6.7	9.7	58	4.15	3.1	5.4		179 (1.64%)	6.19	5.3	7.2
70–74	325	23.94	21.4	26.7	212	17	14.8	19.5		537 (4.92%)	20.62	18.9	22.4
75–79	777	79.58	74.1	85.4	416	49.7	45	54.7		1193 (10.93%)	65.79	62.1	69.6
80–84	1523	197.5	187.7	207.7	820	136.96	127.7	146.7		2346 (21.50%)	171.26	164.4	178.3
85–89	2072	396.56	379.7	414	1040	311.08	292.5	330.6		3112 (28.51%)	363.21	350.6	376.2
90+	2318	677.96	650.6	706.1	1014	661.28	621.2	703.3		3332 (30.53%)	672.8	650.1	715.6
Age not stated	44				14					66 (0.60%)			
Total	7259				3644				11	10,914			
% of T	66.51%				33.39%								

“*” the data item is disclosive or not sufficiently robust for release.

*95% CI* 95% confidence interval, *AMD* age-related macular degeneration, *CVI* certificate of vision impairment, *IR* incidence rate (per 100,000 population), *N/A* not available.

Overall IR increased as age group increased for each type of AMD (Table 3a, b). The most diagnosed cause of AMD was GA with higher IR compared to other types of AMD for all age groups; the highest IR was seen in those aged 90 years and over in 2016/2017 (293.36 per 100,000) and 2017/2018 (284.10 per 100,000).

**Table 3:** (a) Age distribution and incidence rates of CVI due to AMD by AMD type in England 2016/2017, per 100,000 population. (b) Age distribution and incidence rates of CVI due to AMD by AMD Type in England 2017/2018, per 100,000 population.

Age range (years)	Dry AMD (or GA)		Wet AMD (nAMD)		Mixed AMD		Multiple causes incl. AMD		T2
	*n*	IR	*n*	IR	*n*	IR	*n*	IR	*n*
(a)									
0–49	*	*	*	*	*	*	*	*	32
50–54	*	*	*	*	*	*	*	*	21
55–59	12	0.36	*	*	*	*	11	0.33	32
60–64	45	1.54	19	0.65	14	0.48	22	0.75	101
65–69	88	2.9	62	2.04	25	0.82	28	0.92	204
70–74	227	9.53	172	7.22	74	3.11	63	2.65	538
75–79	467	26	378	21.05	170	9.46	174	9.69	1192
80–84	951	70.69	687	51.06	393	29.21	324	24.08	2355
85–89	1375	163.65	906	107.83	564	67.13	489	58.2	3337
90+	1431	293.36	878	179.99	528	108.24	535	109.68	3375
Total T	4632		3132		1772		1665		11,215
(b)									
0–49	*	*	*	*	*	*	*	*	21
50–54	*	*	*	*	*	*	*	*	18
55–59	15	0.43	6	0.17	7	0.2	13	0.37	41
60–64	27	0.91	15	0.5	8	0.27	17	0.57	69
65–69	80	2.78	50	1.73	26	0.9	22	0.76	179
70–74	234	8.98	163	6.26	78	3	60	2.3	537
75–79	496	27.35	349	19.24	206	11.34	141	7.78	1193
80–84	956	69.79	682	49.79	402	29.35	306	22.34	2346
85–89	1237	144.37	894	104.34	528	61.62	449	52.4	3112
90+	1407	284.1	847	171.03	555	112.07	523	105.6	3332
Total T	4492		3032		1827		1549		10,914

“*” the data item is disclosive or not sufficiently robust for release

(a) *n* based on given age, sex and AMD type.

(b) T includes unknown AGE.

(c) T2 includes unknown type of AMD.

*AMD* age-related macular degeneration, *CVI* certificate of vision impairment, *GA* geographic atrophy, *IR* incidence rate (per 100,000 population), *nAMD* neurovascular age-related macular degeneration.

### Public health outcomes framework

**Fig. 1:**
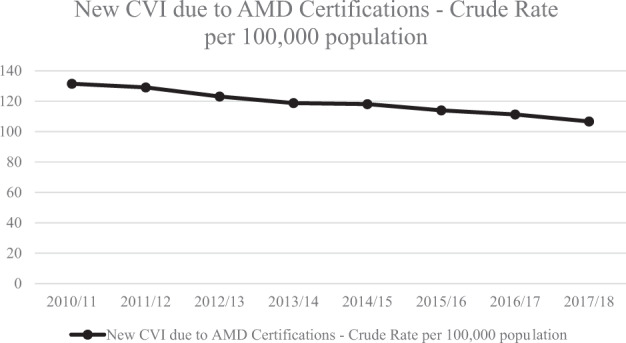
Crude rate trends of new CVI due to AMD in England-PHOF indicator (4.12/E12a). Summary of crude rate trends of new vision impairment certifications due to AMD in people aged 65 and over, in England from 2010 to 2018, per 100,000 population.

PHOF indicator 4.12i/E12a was assessed for trends over the years. The overall rates of AMD certifications have steadily declined in England from 131.5 per 100,000 in 2010/2011 to 106.7 per 100,000 in 2017/2018 (Fig. 1) [17].

**Fig. 2:**
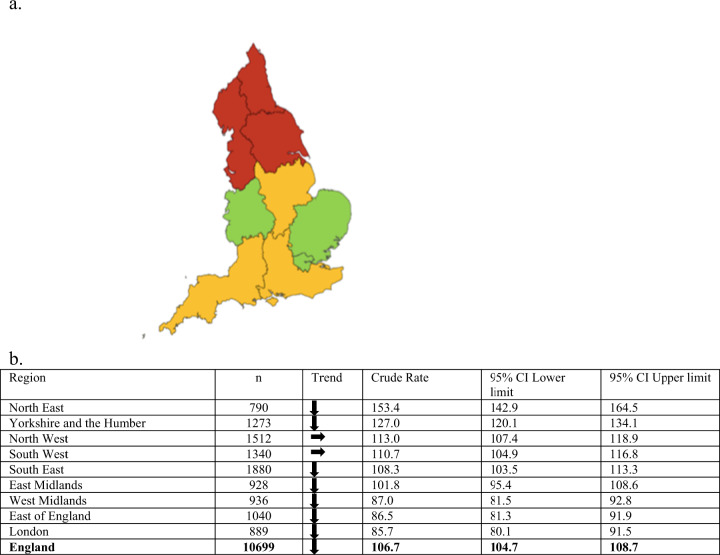
Preventable sight loss certifications due to AMD in England (2017/2018)—PHOF indicator (4.12/E12a). **a** Geographical distribution map of regions for preventable sight loss certifications due to AMD in people aged 65 and over, in England (2017/2018) Crude rates show per 100,000 populations at risk compared with the benchmark for England: green-better, yellow-similar, red-worse. **b** Count and crude rate of preventable sight loss certifications due to AMD in people aged 65 and over, by region in England (2017/2018) Crude rates show per 100,000 populations at risk. Trend: Arrow down—decreasing/getting better, Arrow horizontal—no significant change. 95% CI 95% confidence interval.

Figure 2 shows marked variability between regions in the rates of CVIs due to AMD in those aged 65 and over in England. The northern regions had higher crude rates compared to the benchmark. London had the lowest crude rate (85.7 per 100,000), compared to the North East which had the lowest count but the highest crude rate (153.4 per 100,000) [17].

## Discussion

The findings in this paper were compared to a previous analysis on CVI rates across England between April 2011 and March 2012 by Bunce et al. [3]. In 2011/2012, 23,616 CVIs were collected in total and of those 48.9% (11546) were due to AMD. In this paper 23,453 CVIs were collected in 2016/17 and 22,844 in 2017/2018 with 47.8% due to AMD in both years.

GA accounted for the majority of AMD certifications in the 2011/2012 study and this remained true for 2016/2017 and 2017/2018. The relative contribution of GA decreased from 52.79% (6095) in 2011/2012 to 41.16% in 2017/2018. GA was also shown to have the highest IRs of all types of AMD. The percentages for CVIs due to nAMD and Mixed AMD were relatively unchanged from 2011/2012 to 2017/2018.

There were twice as many women (66%) certified than men (34%) in 2011/2012 and these proportions remained consistent for 2016/2017 and 2017/2018.

In 2011/2012 the largest number of CVIs due to AMD were seen in those aged 85–89 years, with a median age at certification being 86; whereas the age group with the most AMD certifications in 2016/2017 and 2017/2019 were those aged 90 years or older.

The IRs for AMD certifications increased with age in both 2016/2017 and 2017/2018, with the highest IR seen in those aged 90 and over. This trend continued for the different types of AMD and for both sexes.

Only sight loss as a result of nAMD is preventable at this time, as it can be treated to halt or slow the progression of disease [4, 17]. Therefore, the true proportion of current preventable sight loss due to AMD for 2017/2018 is that of nAMD, which is 3032 of the 10,914 CVIs due to AMD, roughly 27.8%.

As demonstrated, there has been an overall decrease in certifications for all types of AMD between 2010/2011 and 2017/2018 (Fig. 1). As treatment is available for nAMD, a reduction in nAMD certifications could be expected; however, growth of the elderly population in England combined with there currently being no treatment available for GA means AMD certification rates should be increasing. This suggests that either reduction in certification reflects a true reduction in the incidence of sight-threatening AMD given the rising number of elderly individuals in England, or not all cases of AMD are being certified and registered with some likely going undiagnosed. This raises questions about the accuracy of true incidence and prevalence estimates of AMD in England.

One possible explanation is that some patients may never seek treatment, attributing sight loss to old age, while others may refuse certification and registration. Hence, these patients will not be included in CVI due to AMD data, resulting in incomplete incidence and prevalence estimates.

A study looking at previous certification for vision impairment, BD8 Certification, found that 51% of those eligible for registration were not certified. The study identified that the main diagnosis of sight loss, in terms of sight impairment status and availability of treatment, was independently associated with non-certification. It estimated that patients with treatable sight impairment were roughly three times less likely to be certified than those where treatment was not indicated [22]. This could explain the decreasing incidence of nAMD. It is important to state that whilst nAMD may be treated effectively, patients may still develop GA [3].

Another explanation for declining certification rates is offered by a study assessing barriers to certification of vision impairment from both patient and health and social care provider perspective [23]. The study reported that long administrative times; lack of clarity on patient pathway, protocol, and processes; plus, difficulty in assessing appropriate timing of certification by ophthalmologists all contributed to poor rates of certification.

The findings in this paper of IRs of AMD CVI registrations being more common in women and increasing with age for all types of AMD is in keeping with the existing literature [3, 24, 25]. AMD is shown to be more prevalent in elderly age groups and age is the most strongly associated risk factor [2, 10, 12, 14, 15]. The age-related eye disease study showed increasing age was strongly associated with GA (OR 3.12; 95% CI 1.91–5.07) and nAMD (OR 4.11; 95% CI 3.09–5.45) [25].

Supplemental Table 1 shows the total CVIs and CVIs due to AMD categorised by type of AMD in 2011/2012, 2016/2017, and 2017/2018 in England.

The decreasing IRs for CVIs due to AMD may reflect increasing non-completion of CVIs as opposed to an actual lowering in IRs of AMD. This is a public health concern as CVI data forms provide the only PHOF national data on preventable causes of sight loss due to AMD in England [23]. Hence, coverage and representativeness are important when estimating the incidence and prevalence CVI due to AMD; especially as this informs the coordination of social services for the visually impaired. The RCOphth recommends that all eligible patients, regardless of treatment option or disease status, should be certified.

In tackling these issues, the next step might be to audit certification coverage and processes in hospital eye clinics to ensure that those with visual impairment needs are supported. Increased awareness, education, and support for patients and ophthalmologists may be needed to improve the certification process and keep CVIs timely, accurate, and assured; to that end monetary incentives to ophthalmologist and Eye Clinic Liaison Officers have been offered by some hospital trusts [3].

### Regional variations

The marked regional variability of count and crude rates of CVIs due to AMD in England may be explained by the demographic profile of the regions. North East had the highest crude rate, whilst London had the lowest. ONS regional population structure by age shows that the North East England has one of the highest proportions of elderly adults aged 65 years and over, that are more likely to suffer from AMD, compared to London which has the lowest [17, 18].

However, the figures do not necessarily reflect differences in incidence of sight loss due to AMD. The geographical differences could point to wider determinants of health which were not included in this analysis such as ethnicity, healthcare education, employment, income, social position, and deprivation [26–28]. In addition, regional attitudes may differ towards certification; and with healthcare not being evenly distributed across England, these may be a barrier to access of ophthalmology services for diagnosis and certification [3, 27].

### Strengths and limitations

This study broke down age into quinary age groups (between 50 and 90 years of age), grouped by sex and divided the types of AMD into those treatable and not treatable. This allowed for a more robust analysis and examination of age and sex specific IRs for the different types of AMD. It would be noted that the diagnosis of AMD in this paper is that recorded on the CVI. Patients with recorded AMD may be more likely to have maculopathy that is not age related so some caution is needed in relation to figures below 50 years of age.

The data does not include those undiagnosed or refusing certification and registration. Case ascertainment varies across England with varying diagnostic, assessment, and data collection protocols. All these factors will affect the accuracy and robustness of IR estimates.

## Conclusion and recommendations

This study has highlighted how the current definition and description of PHOF indicator 4.12/E12a can be argued a misnomer, therefore it needs to be updated to differentiate the types of AMD and divide them into truly preventable and non-preventable causes. The true proportion of current preventable sight loss due to AMD for 2017/2018 is that of nAMD, which is roughly 27.8%.

There is no cure for AMD currently. Therefore, identifying and minimising risk factors combined with early detection and treatment of nAMD is essential.

The reduction in the number of reported CVIs due to AMD over the past decade, despite the ageing and growing populations in England and lack of treatment for GA, suggests that people are either remaining undiagnosed or not being certified or registered. This unmet need is a public health concern and suggests that ophthalmology services may be disjointed and fragmented. There is a need for integrated care pathways for patients with vision impairment involving primary, secondary, and social care. This will facilitate better coordination and continuity of care, which is especially important as patients treated for nAMD may subsequently develop GA. Furthermore, vision health surveillance and screening in those aged 65 and over may improve certification rates for more accurate incidence and prevalence rates.

### Summary table

#### What was known before

- In England, registration for sight loss is achieved following diagnosis and completion of a Certificate of Vision Impairment (CVI) form by a consultant ophthalmologist. Patients are certified by sight impairment status as either sight impaired (SI) or severely sight impaired (SSI). After certification, a copy of the CVI is sent to the Royal College of Ophthalmologists c/o Certifications Office based at Moorfields Eye Hospital, London. This is used for anonymised data analyses, which are incorporated into the Public Health Outcomes Framework (PHOF) by Public Heath England (PHE) under the indicator for preventable sight loss (4.12/E12).
- There are suggestions that CVI and registration data for SI and SSI in England may be incomplete. CVI data only provides incidence of new certifications, not prevalence or incidence of the disease.
- Local Authority register data for blind or partially sighted people does not include cause of sight loss and excludes those who choose not to be registered or those with AMD that do not qualify for registration.
- The actual burden of disease may therefore be underestimated when using CVI data alone, or when supplemented with blind or partially sighted registration data.

#### What this study adds

- This paper provides an update to the data collected by Bunce et al. and details the number of people certified together with incidence rates for the various types of AMD by: sex, sight impairment status, and for all ages using the 2016/2017 and 2017/2018 CVI due to AMD data for England from the Moorfields Eye Hospital, supplemented with 2017–2018 PHOF indicator 4.12i/E12a data.
- The actual incidence rate of preventable sight loss due to AMD is postulated by calculating the incidence rate of nAMD for 2017/2018.
- The PHOF data is used to examine trends over the years and regional variability across England in certification rates for AMD.

## Disclaimer

The data provided by the Royal College of Ophthalmologists c/o Certifications Office based at the Moorfields Eye Hospital captured by the Certificate of Vision Impairment are Department of Health and Social Care copyright. This work was made possible by collaboration with the Royal College of Ophthalmologists. Any views expressed in this publication/document are those of the authors alone and not necessarily those of the Department of Health and Social Care.

## Supplementary information

Supplemental Table 1:
