# Supplementary material for: Is preventable sight loss truly preventable? An exploration of a public health indicator for sight loss due to age-related macular degeneration in England
Source: Eye (Lond). 2022 Feb 23;37(3):516–23. doi: 10.1038/s41433-022-01933-7 (PMC9905582; doi:10.1038/s41433-022-01933-7)
Supplement: Supplementary file 1 — Supplemental Table 1: [file 41433_2022_1933_MOESM1_ESM.pdf]

Supplemental Information: Total CVIs and CVIs due to AMD categorized by type of AMD in 2011/2012, 2016/2017, and 2017/2018 in England.

	2011/2012	2016/2017	2017/2018
Total CVIs	23616	23453	22844
Total AMD (T)	11546 (48.89%)	11215 (47.81%)	10914 (47.78%)
Total Dry GA (T)	6095 (52.79%)	4632 (41.30%)	4492 (41.16%)
Total Wet nAMD (T)	3597 (31.15%)	3132 (27.93%)	3032 (27.78%)
Total Mixed AMD (T)	1820 (15.76%)	1772 (15.80%)	1827 (16.74%)
Total Multiple causes incl. AMD	*	1665 (14.85%)	1549 (14.19%)

"" the data item is disclosive or not sufficiently robust for release*

Key:

AMD – Age-related Macular Degeneration

CVI – Certificate of Vision Impairment

GA-Geographic Atrophy

nAMD -Neurovascular age-related Macular Degeneration
